# CRISPR Genome Editing Applied to the Pathogenic Retrovirus HTLV-1

**DOI:** 10.3389/fcimb.2020.580371

**Published:** 2020-12-23

**Authors:** Amanda R. Panfil, Patrick L. Green, Kristine E. Yoder

**Affiliations:** ^1^ Department of Veterinary Biosciences, College of Veterinary Medicine, The Ohio State University, Columbus, OH, United States; ^2^ Center for Retrovirus Research, The Ohio State University, Columbus, OH, United States; ^3^ Department of Cancer Biology and Genetics, College of Medicine, The Ohio State University, Columbus, OH, United States

**Keywords:** human T-cell leukemia virus type 1, CRISPR, retrovirus, Tax, Hbz, long terminal repeat, pathogenesis

## Abstract

CRISPR editing of retroviral proviruses has been limited to HIV-1. We propose human T-cell leukemia virus type 1 (HTLV-1) as an excellent model to advance CRISPR/Cas9 genome editing technologies against actively expressing and latent retroviral proviruses. HTLV-1 is a tumorigenic human retrovirus responsible for the development of both leukemia/lymphoma (ATL) and a neurological disease (HAM/TSP). The virus immortalizes and persists in CD4^+^ T lymphocytes that survive for the lifetime of the host. The most important drivers of HTLV-1-mediated transformation and proliferation are the *tax* and *hbz* viral genes. *Tax*, transcribed from the plus-sense or genome strand, is essential for *de novo* infection and cellular immortalization. *Hbz*, transcribed from the minus-strand, supports proliferation and survival of infected cells in both its protein and mRNA forms. Abrogating the function or expression of *tax* and/or *hbz* by genome editing and mutagenic double-strand break repair may disable HTLV-1-infected cell growth/survival and prevent immune modulatory effects and ultimately HTLV-1-associated disease. In addition, the HTLV-1 viral genome is highly conserved with remarkable sequence homogeneity, both within the same host and even among different HTLV isolates. This offers more focused guide RNA targeting. In addition, there are several well-established animal models for studying HTLV-1 infection *in vivo* as well as cell immortalization *in vitro*. Therefore, studies with HTLV-1 may provide a better basis to assess and advance *in vivo* genome editing against retroviral infections.

## Introduction

Human T-cell leukemia virus type 1 (HTLV-1) is an oncogenic human retrovirus that transforms CD4^+^ T-cells and causes a variety of diseases including adult T-cell leukemia/lymphoma (ATL) and a neurodegenerative disease called HTLV-1-associated myelopathy/tropical spastic paraparesis (HAM/TSP) (Uchiyama et al., 1977; Poiesz et al., 1980; Yoshida et al., 1982; Gessain et al., 1985; Osame et al., 1986). Despite advances in the field, several unknowns remain regarding HTLV-1-mediated disease development, disease progression, and the lack of effective treatment options. Although HTLV-1 encodes several accessory genes important in the viral life cycle, the two viral proteins which are essential to the pathophysiology of ATL and HAM/TSP are Tax and Hbz. Tax is required for *de novo* infection and cellular immortalization (Bex and Gaynor, 1998; Grassmann et al., 2005), while Hbz supports the proliferation and survival of the infected cell (Arnold et al., 2006; Arnold et al., 2008). Based on a large body of research, targeting these genes will hinder HTLV-1-infected cell growth or survival. To date, CRISPR editing of retroviral proviruses has been largely limited to HIV-1 (Ebina et al., 2013; Rihn et al., 2013; Hu et al., 2014; Liao et al., 2015; Rihn et al., 2015; Zhu et al., 2015; Kaminski et al., 2016; Yoder and Bundschuh, 2016; Yin et al., 2017; Lebbink et al., 2017; Ophinni et al., 2018; Yin et al., 2018; Wang et al., 2018; Darcis et al., 2019; Yoder, 2019). Several detailed reviews of CRISPR gene editing to target HIV-1 have been previously published (Deng et al., 2018; Panfil et al., 2018; Das et al., 2019), and therefore will not be discussed herein. In this review, we explore the use of CRISPR gene editing to disable HTLV-1 and prevent or treat HTLV-1-associated disease.

## Structure of HTLV-1 Genome

**Figure 1:**
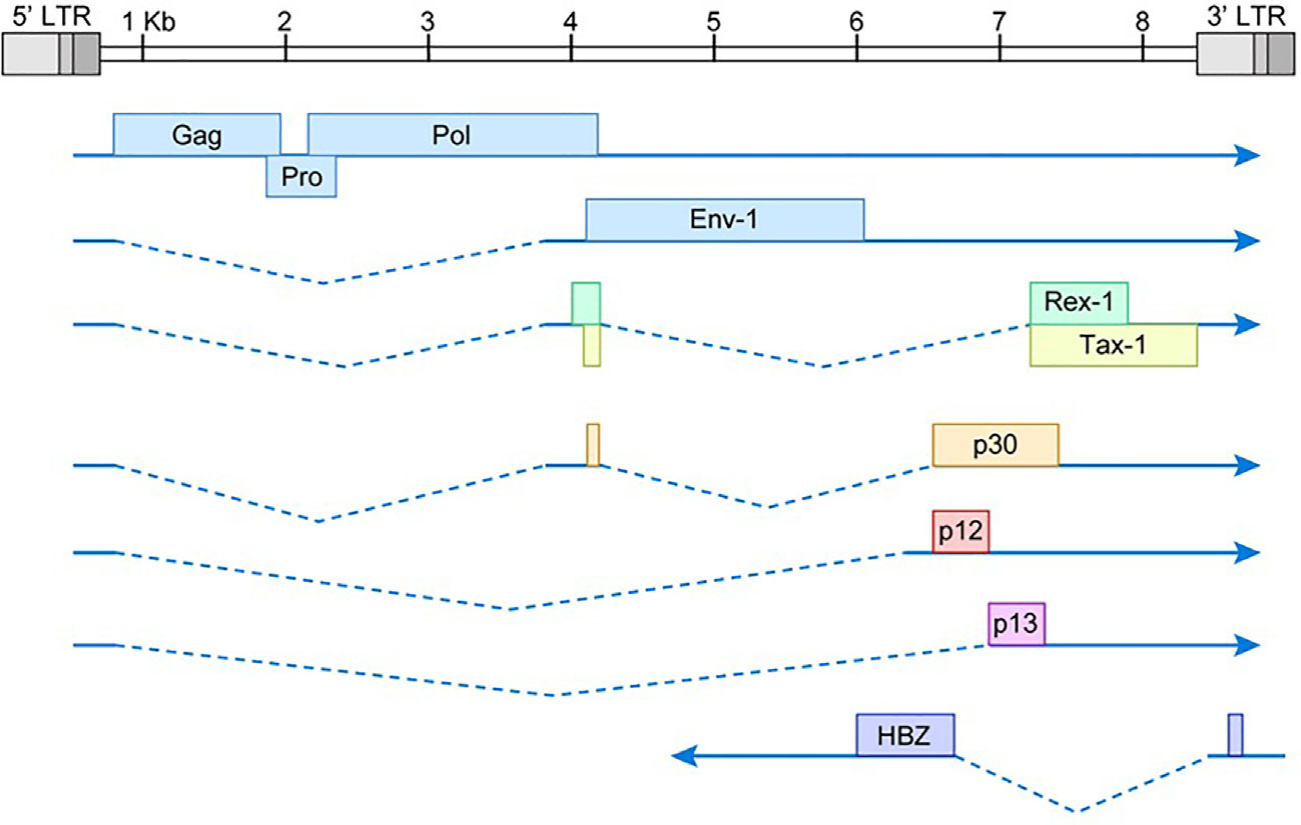
Schematic of HTLV-1 proviral genome. The viral *gag, pro, pol*, and *env* structural/enzymatic genes are flanked by 5’ and 3’ LTRs. The pX region at the 3’ end of the viral genome contains several regulatory and accessory genes. The dotted lines represent spliced regions of each gene product. Drawing is intended to be illustrative and not to exact scale.

HTLV-1 is a complex deltaretrovirus that contains the common retroviral structural and enzymatic genes; *gag*, *pro*, *pol*, and *env* (**Figure 1**). There is also a unique region in the 3’ end of the integrated proviral genome. This region was originally termed ‘pX’ and it encodes several regulatory and accessory genes on both the sense and antisense genomic strands. The viral gene *Tax* is located within the pX region. Tax is encoded by a doubly spliced mRNA with transcription initiating in the 5’ LTR and terminating within the 3’ LTR. Hbz is located on the antisense strand of the proviral genome within the pX and env regions. Hbz is encoded by a singly spliced mRNA with transcription initiating in the 3’ LTR. The HTLV-1 proviral genome is roughly 9kb in length and is flanked by 5’ and 3’ long terminal repeats (LTRs). The LTRs are exact duplicates which consist of a U3, R, and U5 region. These regions facilitate viral integration into the host genome and contain promoter elements, polyadenylation signal sequences, and other regulatory sequences necessary for proper viral transcription.

## HTLV-1 Infection, Persistence, and Disease

HTLV-1 infects an estimated 5–10 million individuals and is found in areas of endemic infection worldwide (Gessain and Cassar, 2012). Regions with prevalent infection include Southwestern Japanese archipelago, parts of the Caribbean, foci in South America, areas in intertropical Africa, the middle East, clusters in Australo-Melanesia, and Romania. Unfortunately, HTLV-1 infection rate is based strictly on reliable epidemiologic data of people in HTLV-1 endemic areas. Consequently, the infection rate of HTLV-1 is estimated to be much higher, since epidemiological data is lacking from several more densely populated areas of the world (Martin et al., 2018).

HTLV-1 can infect a wide range of human cell types, including CD4^+^ T cells, CD8^+^ T cells, B cells, dendritic cells, monocytes, and macrophages (Franchini et al., 1985; Ghez et al., 2006; Furuta et al., 2017). However, HTLV-1 is considered a T-cell tropic virus as it is predominantly found in CD4^+^ T cells *in vivo (* Panfil et al., 2016; Enose-Akahata et al., 2017). This distinct tropism is not at the level of viral entry, but is instead the result of post-infection T cell proliferation and clonal expansion of virally infected CD4^+^ T cells (Kannian et al., 2012). Viral transmission primarily occurs in a cell-to-cell mediated fashion, with cell-free viral infection extremely ineffective (Fan et al., 1992; Pique and Jones, 2012; Alais et al., 2015). Due to the nature of reverse transcriptase, retroviruses like HIV-1 are generally genetically unstable. Remarkably, the HTLV-1 genome is genetically stable and this stability is mostly due to viral amplification that occurs via clonal expansion of infected cells vs. viral replication and subsequent new infections (which is the case for HIV-1) (Furukawa et al., 1992; Wattel et al., 1995; Gillet et al., 2011; Kulkarni and Bangham, 2018). As a result, HTLV-1 has relatively low sequence variation.

HTLV-1 is the etiologic infectious agent of both adult T-cell leukemia/lymphoma (ATL), an aggressive and fatal disease of CD4^+^ T cells (Uchiyama et al., 1977; Poiesz et al., 1980; Yoshida et al., 1982), and HTLV-1-associated myelopathy/tropical spastic paraparesis (HAM/TSP), a chronic inflammatory disease of the central nervous system (CNS) (Gessain et al., 1985; Osame et al., 1986). The incidence of disease related to HTLV-1 infection is 5–10% and occurs after an extensive asymptomatic clinical latency period of up to several decades. The current treatment strategy for ATL varies depending on the severity of the disease and the geographical region. Ultimately, ATL is chemotherapy-resistant and patients consistently relapse (Utsunomiya et al., 2015; Yves et al., 2015). Disease development of HAM/TSP can progress slowly or rapidly, without remission (Matsuura et al., 2016), and is caused by persistent immune activation against proliferating HTLV-1-infected T-cells that infiltrate the CNS. Although there is no cure for HAM/TSP, a number of treatments are available to target pain or inflammation (Enose-Akahata et al., 2017). A recent uncontrolled, phase 1–2a study in Japan suggested the use of mogamulizumab (an anti-CCR4 monoclonal antibody) decreased the number of HTLV-1-infected cells and the levels of inflammatory markers in HAM/TSP patients (Sato et al., 2018). Overall, the precise details of HTLV-1-associated disease development remain poorly defined. However, several studies have shown at least two viral genes, *Tax* and *Hbz*, play a critical role in infection, persistence, and disease development (Matsuoka and Green, 2009; Cheng et al., 2012; Andrade et al., 2013; Enose-Akahata et al., 2017). Therapies that control the expression of HTLV-1 gene products represent a potential treatment for preventing and treating both ATL and HAM/TSP.

The oncoprotein Tax acts as a viral transcriptional activator of both HTLV-1 gene expression (through activation of the viral LTR) and various cellular signaling pathways such as the CREB, NF-κB, and AP-1 pathways (Bex and Gaynor, 1998; Grassmann et al., 2005). Aberrant activation of these signaling pathways helps drive clonal proliferation and survival of HTLV-1-infected CD4^+^ T cells. Tax also causes deregulation of the cell cycle by silencing cellular checkpoints that guard against DNA structural damage and abnormal chromosomal segregation, thus leading to the accumulation of mutations in HTLV-1 infected cells (Arima and Tei, 2001; Giam and Semmes, 2016). Many of the transcriptional effects of Tax, such as LTR activation and NF-κB activation, can be counteracted by the viral protein Hbz (Gaudray et al., 2002; Lemasson et al., 2007; Clerc et al., 2008). Hbz also plays a vital role in regulating genomic integrity, apoptosis, autophagy, and escape from the host immune system surveilance (Matsuoka and Mesnard, 2020). Somewhat surprisingly, Hbz promotes cell proliferation through both its mRNA and protein forms (Mitobe et al., 2015). This accumulating evidence implies that in addition to the viral oncoprotein Tax, Hbz plays a critical role throughout the course of HTLV-1-mediated oncogenesis. It also suggests the balance between Tax and Hbz expression helps determine the outcome of HTLV-1 infection.

HTLV-1 persists *in vivo* in approximately 10^3^ to 10^6^ clones of T cells that survive for the lifetime of the infected host (Bangham et al., 2019). Originally believed to be transcriptionally silent, accumulating evidence suggests the virus is not constantly latent *in vivo*. Recent studies have shown the plus strand of the proviral genome (i.e. Tax) is transcribed in intense, intermittent bursts triggered by cellular stress and modulated by hypoxia and glycolysis (Billman et al., 2017; Kulkarni et al., 2017). These studies also found the minus-strand (i.e. Hbz) is transcribed at lower, more constant levels and is silent in a proportion of cells at given times (Miura et al., 2019). This data supports the observation of persistently activated cytotoxic T lymphocytes (CTLs) directed against plus strand viral antigens. This would suggest that both Tax and Hbz are present (albeit at varying levels and times) in asymptomatic HTLV-1-infected individuals.

Inarguably one of the most important viral regulatory proteins for HTLV-1, Tax oncoprotein expression is typically low or undetectable in most ATL cells (Furukawa et al., 2001; Koiwa et al., 2002; Takeda et al., 2004). Recently however, Tax was found to be expressed in a minor fraction of leukemic cells at any given time, and this expression was spontaneously switched between ‘on’ and ‘off’ states (Mahgoub et al., 2018). This study was performed using the MT-1 cell line and this transient Tax expression is critical for maintaining the infected cell population through activation of anti-apoptotic machinery which persists even after Tax expression is lost. Conversely, Hbz is the only viral gene that remains intact and is consistently found in all ATL cases (Satou et al., 2006). This suggests Hbz expression supports infected cell survival and ultimately, leukemogenesis. Work from our group has shown that shRNA-mediated Hbz knockdown in leukemic cells correlated with a significant decrease in T cell proliferation in culture (Arnold et al., 2008). Engraftment of these leukemic cells in NOD.Cg-PrkdcSCIDIL2rgtm1Wjl/SzJ (NOG) mice will form solid tumors that also infiltrate multiple tissues. When Hbz is knocked down, tumor formation and organ infiltration is significantly decreased compared to animals inoculated with wild-type cells. This data confirms Hbz expression enhances the proliferative capacity of HTLV-1-infected T cells and plays a critical role in cell survival and tumorigenesis.

In HAM/TSP patients CD4^+^CD25^+^ T cells are the main reservoir for HTLV-1, with elevated proviral load strongly correlated with disease pathogenesis (Nagai et al., 1998; Enose-Akahata et al., 2017). Tax mRNA and protein are rarely detectable or below the limit of detection in fresh uncultured PBMCs of HAM/TSP patients. However, Tax mRNA is detected in cells isolated from the spinal cord and cerebellar sections, while Tax protein is detected in the cerebral spinal fluid (CSF) cells of HAM/TSP patients (Lehky et al., 1995; Moritoyo et al., 1999; Cartier and Ramirez, 2005). The chronic presence of Tax in the CSF is thought to induce direct cell damage, such as axonal degeneration in the CNS. The expression of Tax also directly contributes to lymphocyte activation and immunopathogenesis in HAM/TSP (Andrade et al., 2013). Hbz mRNA is detected in PBMCs from HAM/TSP patients, but the transcript level is significantly lower than in ATL patients (Saito et al., 2009). However, the level of Hbz mRNA does appear to correlate with proviral load and HAM/TSP disease severity. Similar immunological features of HAM/TSP have been demonstrated in Hbz transgenic mice (Satou et al., 2011), again supporting a role for Hbz in HAM/TSP disease pathology.

## Gene Editing to Disable HTLV-1

Clustered regularly interspersed short palindromic repeat (CRISPR)/Cas9 genome editing is a relatively new technology that utilizes a guide RNA (gRNA) to target a site-specific DNA double strand break (DSB) by the Cas9 endonuclease. In human cells, DSBs are largely repaired by the error prone non-homologous end-joining pathway, which typically introduces insertions and deletions at the repair junction. Error-prone DSB repair can alter the reading frame of genes, disrupt DNA regulatory motifs, or disrupt the structures of encoded RNA elements. CRISPR is an innovative and powerful genome editing technology that has the potential for development as an HTLV-1 disease therapeutic strategy. In a 2013 report, zinc finger nucleases (ZFNs) that specifically recognized the HTLV-1 LTRs were utilized to disrupt LTR promoter function and inhibit the proliferation of HTLV-1-positive cell lines (Tanaka et al., 2013). This study positively supports the use of genome editing for HTLV-1-infected cells. However, CRISPR/Cas9 technology offers several advantages over ZFNs and transcription activator-like effector nucleases (TALENs) including simplicity, cost effectiveness, and efficiency. Strong support for CRISPR/Cas9 genome targeting of HTLV-1 was also recently reported in 2018. Nakagawa et al. used two different gRNAs targeting Hbz and found ATL cell proliferation was reduced *in vitro (* Nakagawa et al., 2018).

To date CRISPR editing of retroviral proviruses has been largely limited to HIV-1 (Ebina et al., 2013; Rihn et al., 2013; Hu et al., 2014; Liao et al., 2015; Rihn et al., 2015; Zhu et al., 2015; Kaminski et al., 2016; Yoder and Bundschuh, 2016; Yin et al., 2017; Lebbink et al., 2017; Ophinni et al., 2018; Yin et al., 2018; Wang et al., 2018; Darcis et al., 2019; Yoder, 2019). In contrast, HTLV-1 offers more focused gRNA targeting because the viral genome is highly conserved with remarkable sequence homogeneity, both within the same host and even among different HTLV isolates. CRISPR/Cas9 also offers the benefit of being able to disable both latent and actively replicating HTLV-1. The most important drivers of HTLV-1-mediated transformation and proliferation are the *Tax* and *Hbz* genes (Giam and Semmes, 2016; Enose-Akahata et al., 2017; Matsuoka and Mesnard, 2020). Abrogating the function or expression of *Tax* and/or *Hbz* by genome editing and mutagenic DSB repair may disable HTLV-1-infected cell growth/survival and prevent immune modulatory effects and ultimately HTLV-1-associated disease. The viral LTRs are involved in integration of the viral genome into the host chromatin and also serve as promoters to drive expression of all viral genes. These three target regions (Tax, Hbz, LTR) would have the potential to effectively treat newly HTLV-1-infected individuals, asymptomatic viral carriers, and ATL and HAM/TSP patients. Also, given the nature of over-lapping reading frames between Hbz and the 3’LTR, and Tax and the 3’LTR, one can carefully design gRNAs that disrupt two viral elements at once.

## *In Vitro* and *In Vivo* HTLV-1 Models

Several *in vitro* and *in vivo* models exist to study HTLV-1 immortalization, persistence, and tumorigenesis. Importantly, *in vivo* animal models could provide a system to eventually test delivery of CRISPR/Cas9 gene editing components in an animal model with known, effective, and measurable gRNA-viral targets. The different types of *in vitro* and *in vivo* HTLV-1 models are described below:

HTLV has the ability to transform primary T-cells *in vitro* using co-culture immortalization assays (Anderson et al., 2004). HTLV-1 predominantly transforms CD4^+^ T-cells using this technique – identical to what is observed in HTLV-infected asymptomatic individuals and HTLV-1-mediated disease. Because cell-free infection by HTLV is inefficient, *in vitro* infection and immortalization requires irradiated HTLV producer cells co-cultured with freshly isolated peripheral blood mononuclear cells (PBMCs). The initiation of immortalization/transformation is apparent within 5–6 weeks following co-culture as detected by expansion of cells from the peripheral blood lymphocyte mixed cell population. This technique has been extremely useful for examining the requirement of specific gene products on HTLV-1-mediated immortalization in the absence of a functional immune system (Ye et al., 2003; Anderson et al., 2004; Younis et al., 2005; Arnold et al., 2006; Xie et al., 2006; Kannian et al., 2012; Martinez et al., 2019).

NOD.Cg-PrkdcSCIDIL2rgtm1Wjl/SzJ (NOG) mice inoculated subcutaneously with HTLV-1-infected cell lines (Hut-102, SLB-1, ATL-ED, TL-Om1) will develop tumors (Dewan et al., 2003; Ohsugi et al., 2005; Arnold et al., 2008). The transplanted HTLV-1-infected cell lines will also secrete human IL-2Rα, which can be used as a biomarker for cellular proliferation *in vivo*. This allows for simultaneous measurement of tumor formation and growth along with cellular proliferation *in vivo*. Previously, this mouse model has been successfully used by our lab to show that shRNA knockdown of Hbz decreases proliferation of HTLV-1-infected cell *in vivo (* Arnold et al., 2008). The decrease in proliferation *in vivo* correlated with a decrease in tumor size and infiltration of tumor cells to surrounding tissue.

Humanized immune system (HIS) mice model T-cell tropism and lymphoproliferative disease after HTLV-1 infection. The HIS mice are created by injecting human umbilical-cord stem cells into the livers of immunodeficient neonatal NSG mice, resulting in the development of human lymphocytes that appear phenotypically normal but cannot mount an adaptive immune response (Huey and Niewiesk, 2018; Huey et al., 2018). HIS mice inoculated with HTLV-1 consistently reproduce the three key stages of HTLV-1-induced tumorigenesis in a very compact time frame (approximately 4–5 weeks): 1) persistent infection, 2) chronic proliferation of CD4^+^ T-cells, and 3) development of lymphoproliferative disease. Importantly, disease in these mice can be induced using an infectious molecular HTLV-1 clone. Humanized mice can also be generated by intra-bone marrow injection of human CD133^+^ hematopoietic stem cells into NOG mice (Tezuka et al., 2014). Intraperitoneal injection of HTLV-1-transformed cells in these mice also successfully recapitulates ATL disease development.

HTLV-1 infection of rabbits mimic early infection in humans (Arnold et al., 2006; Kannian et al., 2012). Twelve-week old New Zealand white rabbits inoculated with HTLV-1 become persistently infected. The early rabbit humoral antibody responses against Gag and Env mimic asymptomatic early viral infection in humans. These animals do not develop disease, but enable the study of early viral infection events in the presence of a functional immune system. Using infectious molecular HTLV-1 clones, our group has been able to modify the virus to facilitate *in vivo* study of the functional properties of HTLV-1 proteins (Arnold et al., 2006; Kannian et al., 2012; Martinez et al., 2019). Our *in vivo* rabbit model system is advantageous since HTLV-1 long term latency is mediated in part by the host immune response.

## Concluding Remarks

The progression from HTLV-1 infection to disease development (ATL, HAM/TSP) can take up to several decades. Thus, the integrated HTLV-1 proviral genome is clinically latent for long periods of time. The current lack of effective therapies for both ATL and HAM/TSP indicates a need for innovative clinical approaches. HTLV-1 Tax and Hbz are major drivers of transformation, proliferation, and immunological inducing effects. The viral LTRs are also integrally involved in these processes by driving viral transcription and directing integration into the host genome. Targeting any of these viral genes or elements with gene editing would unquestionably alter HTLV-1-infected cell growth/survival and prevent immune modulatory effects and ultimately HTLV-1-associated disease. HTLV-1 is an excellent model to advance genome editing technologies against actively expressing and latent retroviral proviruses. To date CRISPR editing of retroviral proviruses has been limited to HIV-1. In contrast, HTLV-1 offers more focused gRNA targeting because the viral genome is highly conserved with remarkable sequence homogeneity, both within the same host and even among different HTLV isolates. In addition, there are well-established animal models for studying HTLV-1 infection *in vivo* (rabbits, NOG mice, humanized mice) as well as cell immortalization *in vitro*. Studies with HTLV-1 may provide a better basis to assess and advance *in vivo* genome editing against retroviral infections.

## Author Contributions

All authors listed have made a substantial, direct, and intellectual contribution to the work and approved it for publication.

## Funding

This research was supported by NIH R21AI142794 (PG and KY). Additional support was provided by The Ohio State University Comprehensive Cancer Center (AP).

## Conflict of Interest

The authors declare that the research was conducted in the absence of any commercial or financial relationships that could be construed as a potential conflict of interest.
